# Suitability of Different Titanium Dioxide Nanotube Morphologies for Photocatalytic Water Treatment

**DOI:** 10.3390/nano11030708

**Published:** 2021-03-11

**Authors:** Clayton Farrugia, Alessandro Di Mauro, Frederick Lia, Edwin Zammit, Alex Rizzo, Vittorio Privitera, Giuliana Impellizzeri, Maria Antonietta Buccheri, Giancarlo Rappazzo, Maurice Grech, Paul Refalo, Stephen Abela

**Affiliations:** 1 Department of Metallurgy and Materials Engineering, Faculty of Engineering, University of Malta, MSD 2080 Msida, Malta; clayton.farrugia@um.edu.mt (C.F.); maurice.grech@um.edu.mt (M.G.); 2 Consiglio Nazionale delle Ricerche—Institute for Microelectronics and Microsystems, via Santa Sofia 64, 95123 Catania, Italy; alessandro.dimauro@ct.infn.it (A.D.M.); vittorio.privitera@imm.cnr.it (V.P.); giuliana.impellizzeri@ct.infn.it (G.I.); 3 Applied Research & Innovation Centre, Malta College of Arts, Science and Technology, PLA 9032 Paola, Malta; frederick.lia@mcast.edu.mt (F.L.); edwin.zammit@mcast.edu.mt (E.Z.); alex.rizzo@mcast.edu.mt (A.R.); 4 Department of Biological, Geological and Environmental Science, University of Catania, Via Androne, 81-95124 Catania, Italy; mariaantonietta.buccheri@cnr.it (M.A.B.); rappazzo@unict.it (G.R.); 5 Department of Industrial and Manufacturing Engineering, Faculty of Engineering, University of Malta, MSD 2080 Msida, Malta; paul.refalo@um.edu.mt

**Keywords:** titanium dioxide, morphology, water treatment, photocatalysis, nanotubes, anodic oxidation

## Abstract

Photocatalysis has long been touted as one of the most promising technologies for environmental remediation. The ability of photocatalysts to degrade a host of different pollutants, especially recalcitrant molecules, is certainly appealing. Titanium dioxide (TiO_2_) has been used extensively for this purpose. Anodic oxidation allows for the synthesis of a highly ordered nanotubular structure with a high degree of tunability. In this study, a series of TiO_2_ arrays were synthesised using different electrolytes and different potentials. Mixed anatase-rutile photocatalysts with excellent wettability were achieved with all the experimental iterations. Under UVA light, all the materials showed significant photoactivity towards different organic pollutants. The nanotubes synthesised in the ethylene glycol-based electrolyte exhibited the best performance, with near complete degradation of all the pollutants. The antibacterial activity of this same material was similarly high, with extremely low bacterial survival rates. Increasing the voltage resulted in wider and longer nanotubes, characteristics which increase the level of photocatalytic activity. The ease of synthesis coupled with the excellent activity makes this a viable material that can be used in flat-plate reactors and that is suitable for photocatalytic water treatment.

## 1. Introduction

In Europe, 30% of town water supply is used for toilet flushing [1]. This means that water of exceedingly high quality is used in an application where water of a lower quality would suffice. Treated greywater can provide an alternative source of water for toilet flushing. Photocatalytic degradation of the pollutants in greywater and other water matrices allows for effective treatment without the addition of chemicals. Photocatalysis has been shown to be effective at degrading recalcitrant molecules, many of which cannot be degraded in conventional wastewater treatment plants [2,3]. 

TiO_2_ nanoparticles have been used extensively as photocatalysts as they are non-toxic, have a high chemical stability and are resistant to corrosion [4]. Moreover, this wide band-gap semiconductor (≈3.0–3.2 eV) can utilise the UVA portion of the solar spectrum to catalyse the degradation of pollutants [5]. The high oxidative power and large surface area of TiO_2_ nano-powders have been exploited in slurry type photoreactors, where the nanoparticles are suspended in the water being treated [6]. Mounting evidence of the potential harm of TiO_2_ nanoparticles on the environment and the human body makes recovery and responsible disposal of the nanoparticles a priority [7]. Supported catalysts offer an additional advantage in that they eliminate the need for complex separating procedures to remove the nanoparticles after treatment. The anodic deposition of TiO_2_ nanotube layers is a particularly facile production route, as it integrates the synthesis of the photocatalysts and their deposition on a suitable substrate. In anodic oxidation, the nanotube layers are grown from the substrate itself. The anodic nanotube layers have been found to exhibit higher activities compared to immobilised layers of commercially available TiO_2_ powders with the same thickness [8,9,10]. 

Since Zwilling’s first synthesis in 1999, anodic nanotube arrays have been extensively studied [11]. The facile synthetic method is especially attractive since it produces free standing, high aspect ratio ordered layers [12]. The anodic nanotubes are divided into four different generations, where different electrolytes impart different morphological features to the arrays. The first three generations feature the fluoride ions as pitting initiators which, under the influence of an applied potential, result in the progressive evolution from pit to nanotube [13,14]. The use of fluoride ions promotes the production of nanotube morphologies with large diameters and layer thicknesses of several hundred microns [15]. The fourth generation of synthetic methods eschew fluoride ions in favor of chloride ions [16]. In this case, similar morphologies can be attained whilst avoiding the hazards of handling fluoride sources, however, this requires several anodising cycles. The high photocatalytic activity of these materials under artificial UVA light towards the degradation of both biological and chemical contaminants has been amply reported [17,18,19]. The effect of passive irradiation using sunlight as a UV source has also been assessed. It is rather curious that anodic TiO_2_ nanotubes have not been implemented in pilot photocatalytic water treatment reactors. In fact, this facile methodology, the potential for upscaling and the excellent photocatalytic activity provided by these materials remain unexploited [20]. A small-scale solar reactor was produced by Gomes and sunlight’s ability to degrade parabens has been found to be superior to UVA blacklights [21]. McMichael used anodic TiO_2_ nanotubes to drive a solar photoelectrocatalytic disinfection cell [22]. Kim et al. studied the degradation efficiency of these arrays towards different organic compounds [23]. 

In this work, we assess the suitability of different nanotubular arrays produced using some of the electrolytes and methods previously reported in the literature and provide a direct comparison of their activities. Our work will focus on the ability of these materials to degrade pollutants, representing some of the different classes of chemicals which might be present in greywater. Despite the overall low bacterial load of greywater, our research also looked into the antibacterial activity of the materials. The findings of these experiments can help understand how to best exploit the materials in water treatment reactors operating in batch mode. The most suitable material can then be put forward for upscaling and implementation in water treatment reactors. Two nanotube varieties were synthesised in aqueous electrolytes, namely 1 M phosphoric acid [24,25] and 1 M sodium sulfate [26,27], which will hereafter be referred to as TiO_2_NT-P and TiO_2_NT-S, respectively. The first generation of anodic nanotubes is represented by TiO_2_NT-P, whereas TiO_2_NT-S belongs to the second generation. Titanium dioxide formed in the organic electrolyte ethylene glycol (TiO_2_NT-O) forms part of the third generation fluoride-containing electrolytes [28,29]. The photocatalytic efficiency of these three surfaces was assessed and the most suitable material for water treatment was identified. Methylene blue, sodium dodecyl sulfate and paracetamol were used as representative pollutants. *Escherichia coli* (*E. coli*) was used to quantify the antibacterial activity of the materials. Methylene blue has been used extensively as a model representing dyes that bleed out from garments during laundry and are present in household greywater [30]. Sodium dodecyl sulfate is an anionic surfactant which contributes to the cleaning action of most personal care products. The concentration of the surfactant in wastewater is subject to stringent limits [31]. The presence of paracetamol in surface and shallow ground water has been attributed to irrigation with greywater. Given the ubiquitous nature of paracetamol and the fact that irrigation is one of the potential uses of reclaimed greywater, then further investigations into the potential for its degradation are worthwhile [32,33]. *E. coli* is used as an indicator of faecal contamination and its inactivation by photocatalysis is used to assess the activity of materials for the reclamation of wastewater [34,35]. 

## 2. Methodology

### 2.1. Material Synthesis

Anodising was carried out in a typical two electrode set-up using titanium (99.6%, Grandis, Rancho Santa Margarita, CA, USA) plates. The anode and cathode had identical dimensions and were held 30 mm apart. A Delta Elektronika SM300-20 DC power supply (Delta Elektronica, Zierikzee, The Netherlands) was used. In case of the TiO_2_NT-S, a solution of 0.5 wt% sodium fluoride (Fluka, Charlotte, NC, USA) in 1 M sodium sulfate (Sigma, Gillingham, Dorset, UK) was used as the electrolyte. The synthesis was carried out at 20 V for six hours. The electrolyte used in the production of TiO_2_NT-P comprised a 0.5% wt% sodium fluoride solution in 1 M phosphoric acid (Scharlau, Barcelona, Spain). A 20 V potential was applied for a period of three hours. A solution containing 0.5 wt% ammonium fluoride, 3 wt% water and balance ethylene glycol was the medium used to produce TiO_2_NT-O. A shorter synthesis time of one hour at 70 V was used. In all cases the as-produced nanotube arrays were rinsed briefly in ethanol and annealed for two hours at 450 °C. The annealed samples were sonicated in isopropyl alcohol for five minutes. 

### 2.2. Morphology and Structural Analysis

The morphology of the arrays was studied using a Zeiss Merlin field emission scanning electron microscope (SEM) (Carl Zeiss, Oerzen, Germany). Image analysis was carried out using ImageJ (National Institutes of Health, version 1.8.0_172, image analysis software, Bethesda, MD, USA, 2021). The crystal structure of the catalysts was analysed using a Rigaku Ultima IV Cu-Source X-ray diffractometer (Rigaku Corporation, Tokyo, Japan). 

The wettability of the surface was assessed by measuring the optical contact angle using a Dataphysics OCA 25 system (DataPhysics Instruments GmbH, Filderstadt, Germany) and a drop size of 15 µL. Prior to testing the effect of UV irradiation, the surfaces were cleaned by irradiating the samples for 1 h using a wavelength of 365 nm at 10 mW/cm^2^ as per ISO 10678:2010. This ensured the removal of adsorped hydrocarbons on the surfaces [36]. The samples were then stored overnight in sample bags. The photoinduced changes to the contact angle were measured after irradiation using a wavelength of 365 nm at 10 mW/cm^2^.

### 2.3. Optical Properties

Diffuse reflectance spectra were collected on a Shimadzu SolidSpec-3700 UV-VIS spectrometer, equipped with an integrating sphere (Shimadzu Corporation, Kyoto, Japan). A scan range of 280 to 700 nm was used, sampling was conducted at 1 nm intervals at medium speed. The values were corrected for the solar global tilt as specified in the ASTM G173-03 standard [37]. The total spectral absorptance over the range of interest was calculated using Equation (1).

(1) $$\alpha =\frac{\int_{\lambda_{1}}^{\lambda_{2}}\alpha_{\lambda }G_{\lambda }d\lambda }{\int_{\lambda_{1}}^{\lambda_{2}}G_{\lambda }d\lambda }$$

where *λ* is the wavelength, *α*_λ_ is the absorptance at a specific wavelength, *G*_λ_ is the solar spectral irradiance at a specific wavelength.

### 2.4. Photocatalytic Activity

The degradation of methylene blue under UV light was used to assess the photocatalytic efficiency of the materials. The method employed was an adaptation of ISO 10678:2010 [38]. The samples were initially irradiated using a UWAVE LED system (UWAVE, Les Ulis, Paris, France) with a wavelength of 365 nm and an intensity of 10 mW/cm^2^. This removed any adventitious hydrocarbons from the surface [36]. The samples were then immersed in 10 mL of a 1.5 × 10^−5^ M methylene blue (MB) solution and left for one hour in the dark to reach the absorption—desorption equilibrium. The absorbance of the solution after irradiation was measured at thirty-minute intervals for a period of four hours. A scan range between 500 and 800 nm was used and the absorbance at 664 nm was read on a Perkin Elmer Lambda 35 UV-VIS spectrophotometer (Perkin Elmer, Waltham, MA, USA). 

The materials were also tested for the degradation of paracetamol, one of the most popular analgesic and antipyretic drugs. The measurements were performed with the same procedures described above for the MB photodegradation. A starting solution of 1.5 × 10^−5^ M paracetamol in deionized water was used. The degradation of paracetamol was evaluated by following the absorbance peak at 243 nm in the Beer–Lambert regime [39].

The photocatalytic activity was investigated further by measuring the degradation efficiency of sodium dodecyl sulfate (SDS), which is an anionic surfactant found in most personal hygiene products. The test preparation and irradiation methodology were identical to that employed in the MB tests. The initial concentration of the solution was 3.5 mg/L equivalent to 1.5 × 10^−5^ M. The absorbance of the solutions was measured before and after irradiation with a HACH DR 3900 spectrophotometer using LCK 332 and LCK 432 cuvette kits for high and low concentrations, respectively (HACH, Loveland, CO, USA). The solutions were diluted when necessary.

The MB and paracetamol degradation tests were also conducted using an Oriel VeraSol-2 solar simulator (Newport Corporation, Irving, CA, USA). To investigate dye sensitisation, the MB test was first carried out using the full solar spectrum and then repeated without the 500–700 nm range which contains the absorbance peak of MB. 

The antibacterial activity of the nanotube arrays was assessed by determining the surviving bacteria. A colony of *Escherichia coli* ATCC^®^25922 (American Type Culture Collection, Manassas, VA, USA) was transferred to 100 mL of sterile Luria Bertani (LB) broth (Sigma, Gillingham, Dorset, UK). The inoculum was incubated overnight and held at 37 °C with constant agitation at 180 RPM under aerobic conditions. The optical density at 600 nm was measured the next morning. Suitable 1/10 serial dilutions were prepared from the original culture. A total of 50 µL from the last three dilutions in the series were plated on LB agar plates (Sigma, Gillingham, Dorset, UK) and incubated overnight at 37 °C. A 200 µL aliquot from the second serial dilution was transferred to the photocatalyst, ensuring complete coverage of the surface. In order to avoid evaporation of the bacterial culture, the coupons were covered with a quartz glass, while avoiding contact between the quartz and the bacteria. The photocatalysts were irradiated for one hour with a Philips TL8 black light (Philips Lighting, Eindhoven, The Netherlands) at 3.5 mW/cm^2^ at a wavelength of 365 nm. The photocatalyst was then transferred to a 35 mm petri dish containing 10 mL of phosphate buffered saline. The petri dish was covered, and the contents were shaken for one minute. A 50 µL aliquot from the petri dish was plated on LB agar. Suitable 1:10 dilutions of the bacteria recovered from the surface, were prepared and plated. The plates were then incubated upside down at 37 °C overnight. The colonies were counted the following morning. 

## 3. Results and Discussion

**Figure 1:**
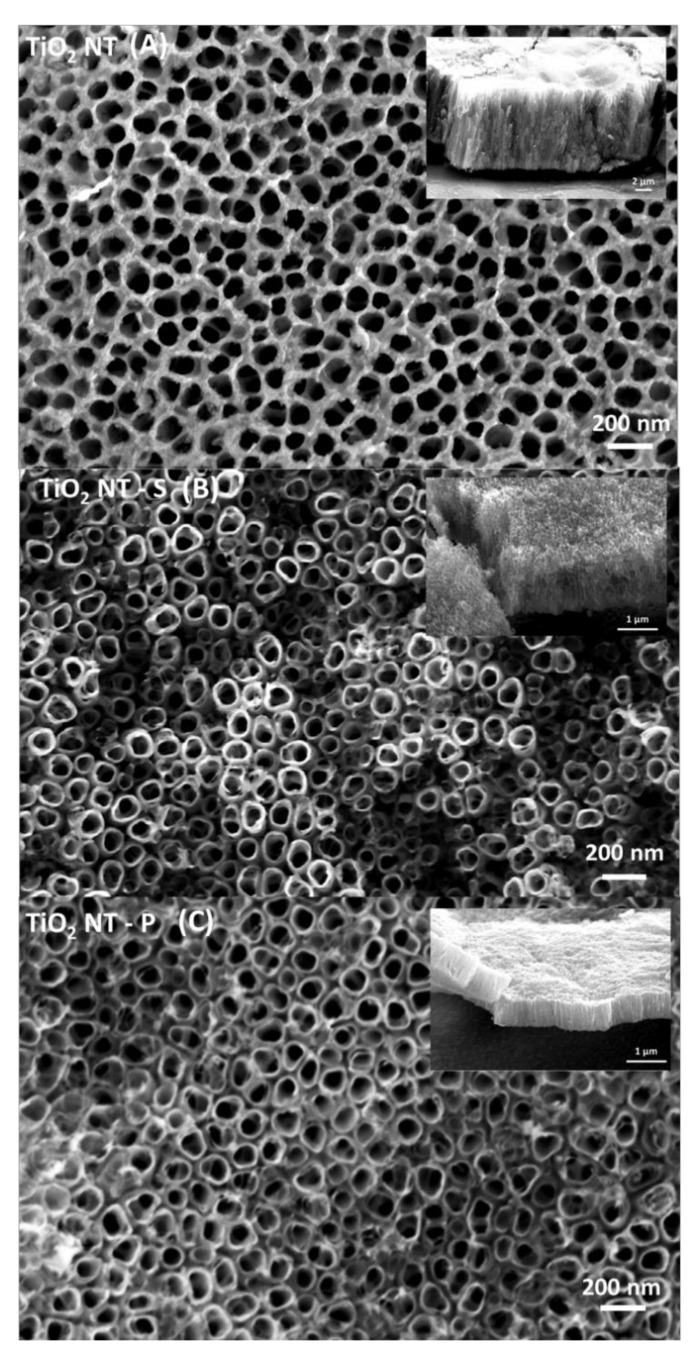
Scanning Electron Microscopy (SEM) image showing the morphology of the different arrays (**A**) TiO_2_NT-O, (**B**) TiO_2_NT-S, (**C**) TiO_2_NT-P. The insets show a side view of a mechanically removed layer.

The morphologies obtained using different synthesis methods are presented in Figure 1A–C. Table 1 summarises the main morphological features of the different nanotube arrays. Free standing nanotubes were formed in all cases, with those for TiO_2_NT-S and TiO_2_NT-P appearing as discrete tubes. The nanotubes in TiO_2_NT-O appear to have no distinct tube diameter due to a superficial TiO_2_ and titanium hydroxide layer (TiOH*_x_*) [40]. This thin oxide film was not completely etched off due to the shorter process time of one hour and could be removed by sonicating the samples for longer. This was avoided in order to decrease the possibility of collapse of the nanometric array at points which are mechanically weaker. The inset in Figure 1A reveals that separate nanotubes were successfully formed. The TiO_2_NT-O array has a layer thickness of 9.99 ± 0.48 µm which is several times thicker than that of those synthesised in the aqueous counter media, clearly showing the effect of the applied potential on the morphology. A higher potential between the anode and cathode produces a stronger electric field which augments the transport of fluoride ions towards the anode surface. These fluoride ions etch through the oxide layer to form the nanotubular structure. Higher potentials result in increased rates of etching, leading to longer tubes and large diameters [41]. High anodisation potentials can only be employed when a non-aqueous electrolyte is used. In aqueous electrolytes voltages exceeding 20 V result in the loss of the nanotubular structure [42,43]. Despite the ability to grow thicker layers in aqueous media, layers exceeding 10 µm tend to have poor adhesive properties, resulting in their delamination [44]. In aqueous electrolytes, the oxidation rate at higher voltages can result in the anodic dissolution of the material rather than the formation and remodelling of an oxide layer. The higher viscosity of the organic electrolyte, increases the resistivity of the anodizing medium, thus allowing for higher voltages to be sustained before the breakdown voltage is exceeded, resulting in the collapse of the nanostructure [45]. The difference in length between TiO_2_NT-S (1.45 ± 0.07 µm) and TiO_2_NT-P (0.676 ± 0.02 µm) is due to the longer duration of the process for TiO_2_NT-S since the voltage was kept constant at 20 V for both processes. Adan et al. reported that longer nanotubes provide a larger area over which light can be absorbed, thus, increasing the overall absorbance enhancing the photocatalytic activity. The higher surface area also increases the amount of pollutant interacting with the photocatalyst [1]. On the other hand, the advantage of longer nanotubes may have limitations. In fact, Marien et al. reported that once a certain length is exceeded, the amount of light reaching the lower portions of the tube is minimal, as these are shadowed. Additionally, as the nanotube length increases, the pollutants need to diffuse over a longer path, resulting in lower activities [46]. One benefit of longer arrays is the possibility of greater longevity and prolonged activity, as breakage of the tube tops would not affect the overall amount of photocatalytic material available. Ultrasonic cleaning to remove surface debris can damage the tube tops causing their eventual breakage,0 especially when this is conducted at low frequencies for extended periods of time [47].

The diameters of the TiO_2_NT-S and TiO_2_NT-P are practically identical. This is to be expected given that the same potential was used in the two processes. On the other hand, the higher voltage used in TiO_2_NT-O resulted in larger pore diameters. The wall thicknesses for the TiO_2_NT-S and TiO_2_NT-P processes are 13.99 ± 2.64 nm and 11.71 ± 2.14 nm, whilst that of TiO_2_NT-O tubes is 10 ± 1.67 nm. Wider nanotubes can facilitate the diffusion of the pollutant into the tube whilst maximising the amount of light absorbed [46]. However, nanotube diameters greater than 100 nm can lead to a decreased activity due to the lower specific surface area of the nanotube. There is, thus, a specific ratio of length to width of the nanotubes which results in the most active material [48]. Given that this ratio is met, shorter and thinner tubes are preferred, as they provide enhanced activity due to the charge carrier separation being high over short diffusion distances [49]. The largest aspect ratio recorded in this study was that for TiO_2_NT-O, followed by that of TiO_2_NT-S and finally, TiO_2_NT-P. The suitability of these morphological parameters was assessed by measuring the photocatalytic activities of the three different materials. 

**Figure 2:**
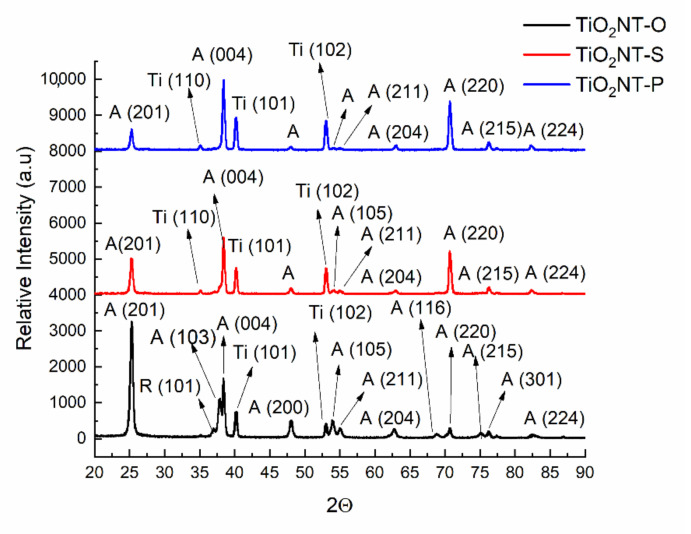
X-Ray Diffraction (XRD) patterns of the three photocatalytic materials. A = anatase, R = rutile, Ti = titanium.

The crystalline structures obtained with the different anodising processes are presented in Figure 2. Anatase and titanium were the main constituents of the three materials. A low intensity rutile shoulder at 37° and another shoulder at 77° were recorded for TiO_2_NT-O. Despite the fact that nanotubes grown in aqueous media and annealed at 450 °C [42] typically contain no rutile, a small rutile peak was observed for TiO_2_NT-P at 77°. Rutile has been reported to have a higher visible light absorbance than anatase, which, on the other hand, has the higher activity in the UV region. A heterostructure composed of the two polymorphs should, thus, have a higher solar photocatalytic activity than the separate phases, as it can utilise a higher percentage of the solar spectrum [50]. The benefits of having rutile in the heterostructure are still not universally acknowledged, as it promotes a fast charge carrier recombination which can diminish the overall efficiency [51]. The peak at 25° for TiO_2_NT-O has the highest intensity, followed by that of TiO_2_NT-S, with the peak for TiO_2_NT-P having the lowest intensity. The different planes of anatase, namely, (103) and (004) were manifested in a bifurcated peak at around 38°. As expected, the thickest layer, TiO_2_NT-O resulted in the lowest substrate detection and consequently the lowest titanium peak at 41°. It appears that the electrolyte results in growth in a specific direction. The [220] direction is preferred when aqueous electrolytes are used and the [201] direction when the organic electrolyte is used. 

**Figure 3:**
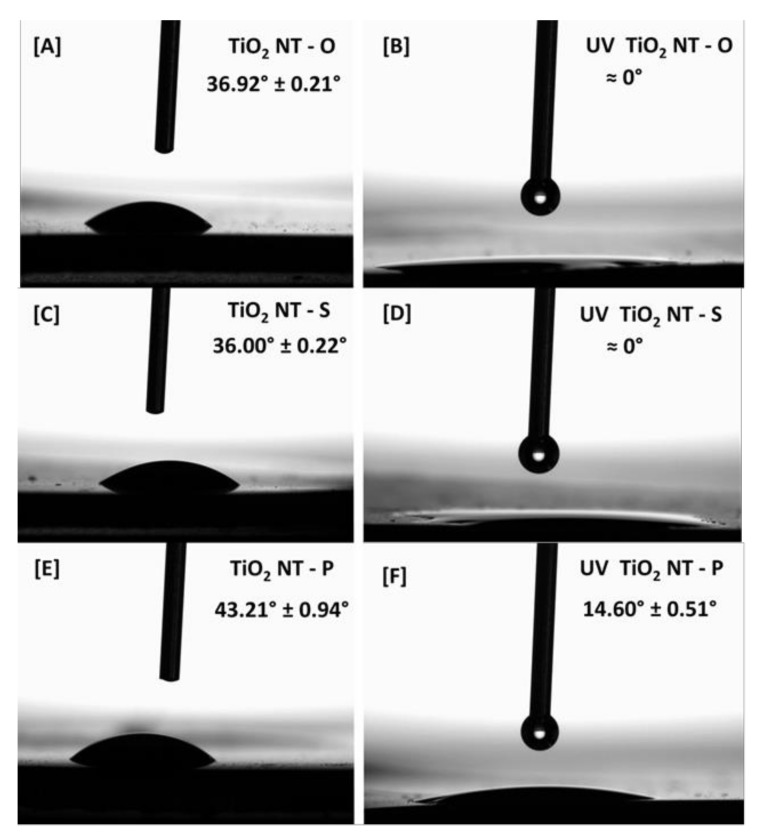
Optical contact angles of the nanotube arrays. (**A**) TiO_2_NT-O without irradiation; (**B**) TiO_2_NT-O with UV irradiation; (**C**) TiO_2_NT-S without irradiation; (**D**) TiO_2_NT-S with irradiation; (**E**) TiO_2_NT-P without irradiation; (**F**) TiO_2_NT-P with UV irradiation.

Good wettability ensures proper contact of the contaminants with the surface, both by reducing diffusion gradients and by ensuring complete coverage of the surface with the water being treated. The collage in Figure 3 presents the contact angle of the materials before and after irradiation with UVA. The nanotubular structure of all three materials resulted in a hydrophilic contact angle. The hydrophilicity of the un-irradiated surfaces is derived from the filling of the nanotubes through wicking of the water from the surface, causing the droplet to spread. A large pore diameter generally improves the wetting behaviour of the surface [52,53]. However, despite having a larger diameter, the contact angle for TiO_2_NT-O at 36.92° (Figure 3A) is marginally greater than that for TiO_2_NT-S, with a value of 36.00° (Figure 3C). The contact angle for TiO_2_NT-P at 43.21° (Figure 3E) is larger than that of the other two materials. The diameters of TiO_2_NT-P and TiO_2_NT-S are similar, highlighting a possible contribution of tube length and orientation to the wettability of the surfaces. 

The effect of UV irradiation on wettability is immediately evident. As can be seen from Figure 3B,D, complete wettability of both TiO_2_NT-O and TiO_2_NT-S surfaces was achieved after one hour of irradiation. This indicates that photo-induced superhydrophilicity has been achieved. In the case of the TiO_2_NT-P surface, (Figure 3F), a reduction in the contact angle to 14.60° was recorded, albeit this was still higher than that of the other materials. Three mechanisms have been put forward to explain similar changes in wettability, namely: the generation of surface vacancies [54], the reconstruction of Ti-OH bonds [55] and the decomposition of adsorped organics [56]. Since all the mechanisms involve the generation of electrons and electron holes, wettability could, thus, be considered as an indicator of photo-induced activity. The lower wettability of TiO_2_NT-P could, thus, indicate a lower overall photocatalytic performance. 

**Figure 4:**
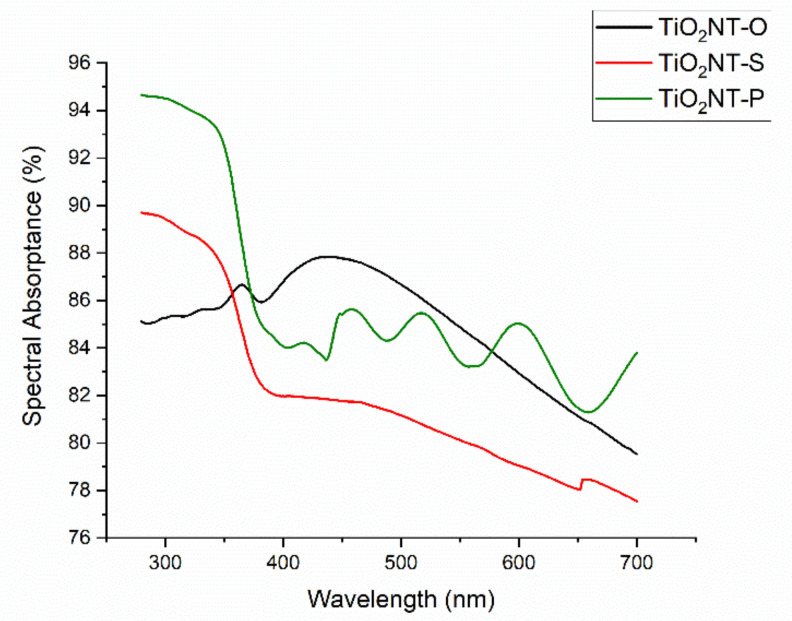
Spectral absorptance of the three nanotube arrays.

The absorptance spectra of the three materials are presented in Figure 4. The spectra for TiO_2_NT-S and TiO_2_NT-P have absorptance maxima in the UVB region. The absorptance values in the UV region of the spectrum of TiO_2_NT-O fluctuate slightly around the 85 to 86% range. The absence of UV absorptance maximum in the TiO_2_NT-O array has been attributed to the TiO_2_ cementing the tubes together possessing a mirror effect [57]. The calculated spectral absorptance values presented in Table 2, indicate that, despite some differences in the spectra, the overall absorptance is quite similar. Similarly, despite the visible portion of the spectra not showing any commonalities, the calculated absorptances are also very close. This might indicate that the optical properties of the materials have little bearing on their photocatalytic activity. For TiO_2_NT-S and TiO_2_NT-O the absorption of visible light decreased with an increase in wavelength. An absorption maximum was recorded for TiO_2_NT-O at 450 nm, which was followed by declining values. Oscillations in the visible region of the TiO_2_NT-P spectrum were recorded. These oscillations have been attributed to interference fringes resulting in thin transparent films [58]. Given that TiO_2_NT-P was the thinnest material and had a translucent appearance, this phenomenon was only recorded in this instance. The total spectral absorption was calculated and tabulated in Table 2. The value for the UV region is of interest given that a high level of absorptance could aid in ensuring a high photocatalytic activity. Although possessing the lowest thickness of the three materials, the absorptance of TiO_2_NT-P at 83.8% was comparable to that of the much thicker TiO_2_NT-O. Given that the morphology of each array is also dependent on the electrolyte, with different structures arising as a result, it is extremely difficult to tie a particular absorptance value to a morphological feature.

**Figure 5:**
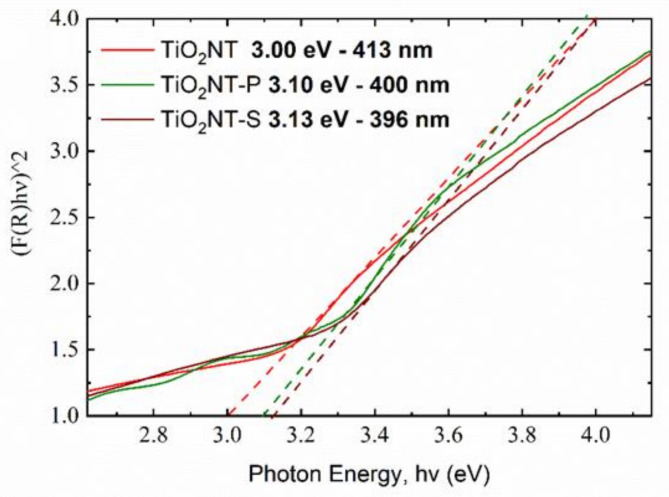
Tauc plots for the different nanotube arrays.

From the diffused reflectance spectra, the band gap of the materials was calculated. The tauc plots in Figure 5 present the band gap of the three materials. When it comes to photocatalysis, lower bandgaps are desired as these indicate the ability of the material to be activated by light of lower energy, i.e., visible light. The band gap of titanium dioxide is 3.2 eV, meaning that wavelengths lower than 387 nm are needed to generate the charge carriers [59]. It can be noted that the bandgaps are slightly lower than that of pristine anatase, and thus, the materials can absorb a small portion of the visible spectrum. The bandgap, however, is not indicative of the overall efficiency of the material, and thus, the rate of visible light activity must be determined experimentally. 

To better understand the photocatalytic activity of the different materials, and therefore, their ability to degrade organic pollutants, MB, SDS and paracetamol were used as probe molecules. These molecules belong to different classes of chemicals, with different molar masses, charges, and chemical structures, and thus, can provide a more holistic idea of the activity of the materials. The solutions without the photocatalysts were irradiated to determine the contribution, if any, of photolysis towards the final degradation rate. The following methods use Beer Lambert’s law to quantify the change in concentration of the target analyte [39]. 

**Figure 6:**
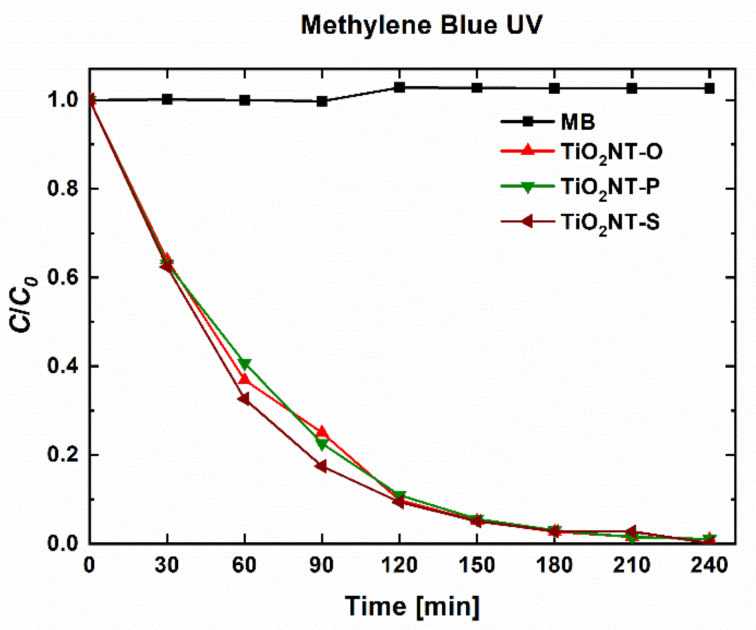
Degradation efficiency of nanotube arrays towards Methylene Blue (MB) after irradiation with UVA light.

A method based on ISO 10678:2010 was used as a preliminary screening test, where the decolourisation of the solutions indicates the photocatalytic activity. The change in MB concentration after irradiation is presented in Figure 6. Complete degradation was achieved by all the three materials. This is in line with the findings of Lin et al., who reported the complete degradation of MB over similar test durations [60]. The effect of morphology on the degradation rate of MB has been studied by Pasikhani et al. [61]. The authors claim that the fastest degradation rate occurred with nanotubes with the largest aspect ratio. 

**Figure 7:**
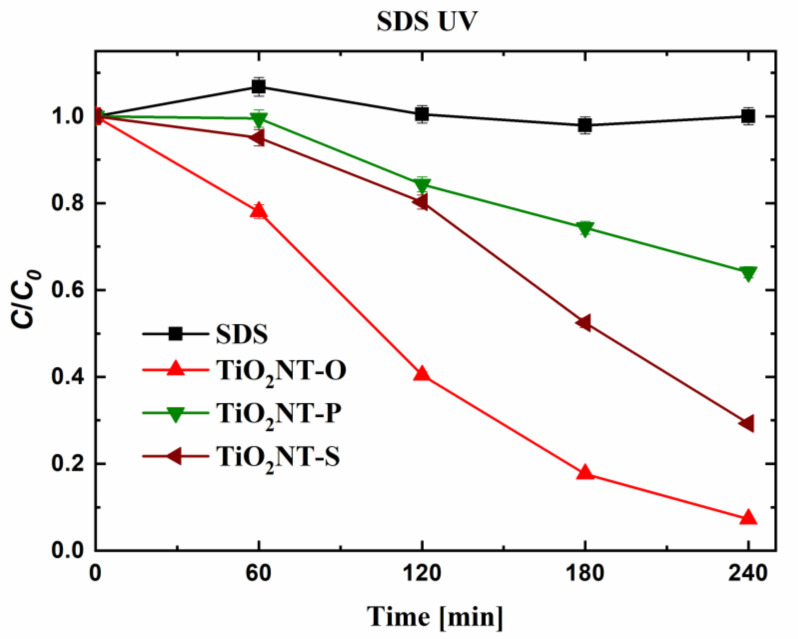
Degradation efficiency of nanotube arrays towards sodium dodecyl sulfate (SDS) after irradiation with UVA light.

The results for the degradation of SDS show a significant deviation from those obtained for methylene blue (Figure 7). In this case, degradation was highest with the TiO_2_NT-O surface, with the initial concentration falling to 6% of the original value, after 4 h. The corresponding values with the TiO_2_NT-S and TiO_2_NT-P surfaces were 29 and 64%, respectively. The molecular weight of MB is 319.85 and the molecule has extensive conjugation resulting in the chromophore which gives it an intense blue colour. SDS has a lower molecular weight at 288.31 and is devoid of a chromophore. The higher degradation potential of the MB might be attributed to the breakage of one of the bonds constituting the chromophore, which will lead to the decolourisation of the solution. Similarly, reduction of the MB molecule from MB^+^ to leuco-methylene blue by photogenerated electrons can also result in decolourisation [62]. On the other hand, the quantification of SDS is less susceptible to a false-positive results, arising from the scission of the molecule. If the resulting residue is still significantly non-polar, it will still migrate to the organic layer upon forming the ion pair with methylene blue. This keeps the absorbance values high. Another factor to be considered is the attraction of the molecules towards the surface. If there are significant repulsive forces, degradation can be inhibited. The lower degradation efficiency of the TiO_2_NT-S and TiO_2_NT-P can be attributed to the difference in thickness of the layers, with the thinner TiO_2_NT-P having the lowest reactivity. Thicker layers can absorb more of the UV radiation, and thus, can sustain higher reaction rates. This relationship holds up to a certain thickness, beyond which, the charge carriers generated deeper in the material can recombine before reaching the surface [1]. Similarly, the material at the nanotube bottom is shadowed if the nanotubes are sufficiently long. 

**Figure 8:**
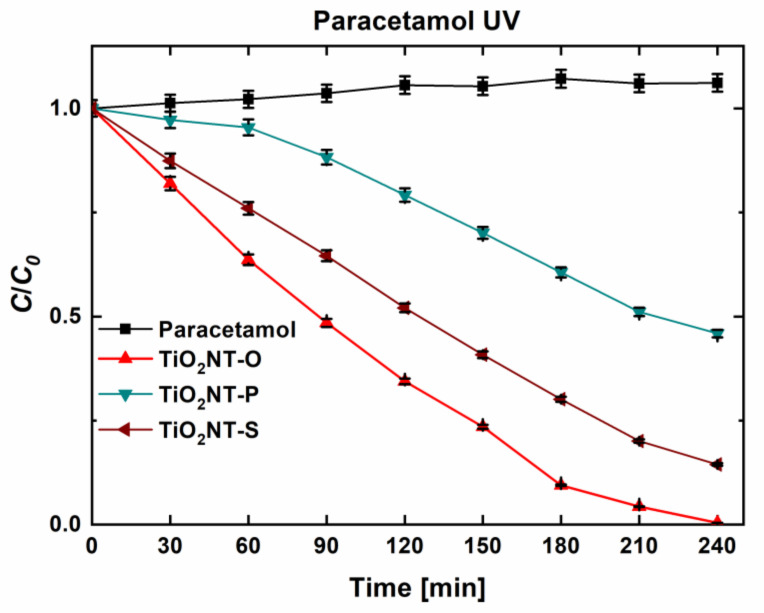
Degradation efficiency of nanotube arrays towards paracetamol after irradiation with UVA light.

As can be seen in Figure 8, a similar trend was observed for the degradation of paracetamol. TiO_2_NT-O was the most effective material, resulting in the complete degradation of the molecule. The TiO_2_NT-S and TiO_2_NT-P materials reduced the initial concentration to 15 and 46%, respectively. In general, all the three materials exhibited a better disposition to degrade the paracetamol rather than the SDS. This indicates that when stable molecules are employed as probe species, the degradation of smaller molecules is favoured. This might be due to a more efficient anchorage of the molecule to the surface. However, despite this, the morphology of the nanotubes, rather than the complexity of the molecules, appears to be the major contributor towards activity. 

**Figure 9:**
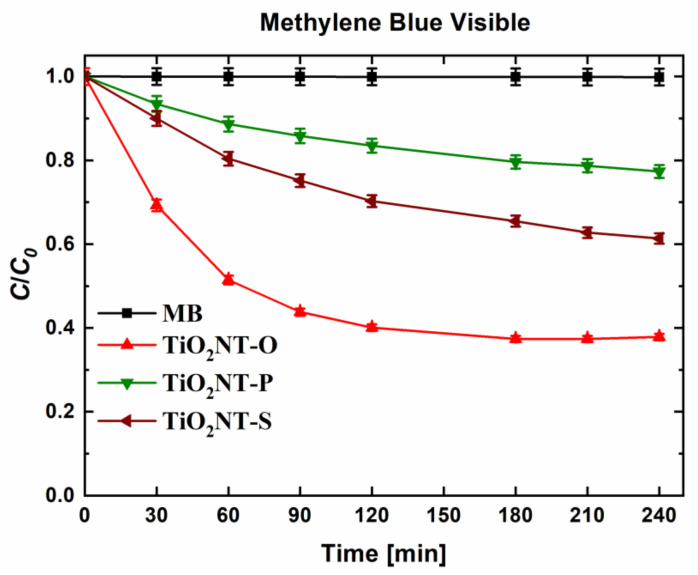
Degradation of methylene blue (MB) by visible light.

**Figure 10:**
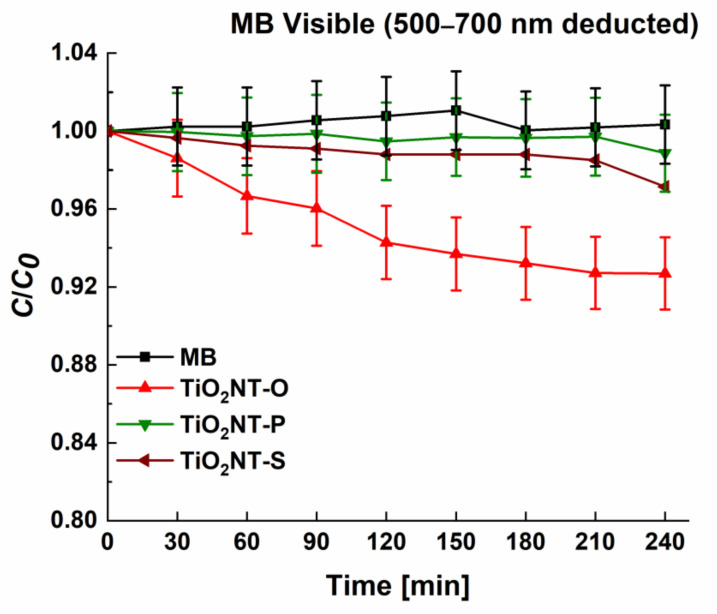
Assessment of dye sensitisation using a reduced spectrum.

The visible light activity of unmodified TiO_2_ nanotubes was determined and described by Valeeva et al. [63]. Given that the bandgaps of the materials indicate some level of visible light absorbance, the photocatalytic activity in visible light was assessed. The testing parameters were kept identical to those employed in the UV degradation of MB. The results in Figure 9 indicate that the degradation of methylene blue can be catalysed by visible light. The trends in activity are the same as for the degradation under UV light, albeit the activity is lower. However, this is true for many visible light active catalysts, where irradiation with light of lower energies lends to lower rates of reaction [64]. A final concentration of 38% was obtained with TiO_2_NT-O with higher values of 61 and 78% for TiO_2_NT-S and TiO_2_NT-P, respectively. The mechanism of the visible light induced degradation was studied in more detail. A scan with wavelengths between 200 and 800 nm showed that the MB molecule has a maximum absorptivity of wavelengths between 500 and 700 nm. By blocking this range of wavelengths, degradation due to dye sensitisation can be determined. Methylene blue and other dyes such as rhodamine b have been used extensively as photosynthetisers [65,66]. The resulting photocatalytic activity by visible light was found to be extremely low, with a maximum value of 8%, achieved by TiO_2_NT-O, followed by TiO_2_NT-S at 3% and TiO_2_NT-P at 1% (Figure 10). This indicates that sensitisation helped bolster the visible light activity. Sensitisation in this case resulted from the experimental procedure employed. The initial equilibration step allows for the adsorption of some of the MB molecules onto the photocatalyst surface [67]. The adsorbed molecules when irradiated by visible light reach their excited state and inject electrons into the conduction band of the photocatalyst [68]. The photosensitiser enhances the effect of visible light, which on its own would not have sufficient energy to allow the transition to occur.

**Figure 11:**
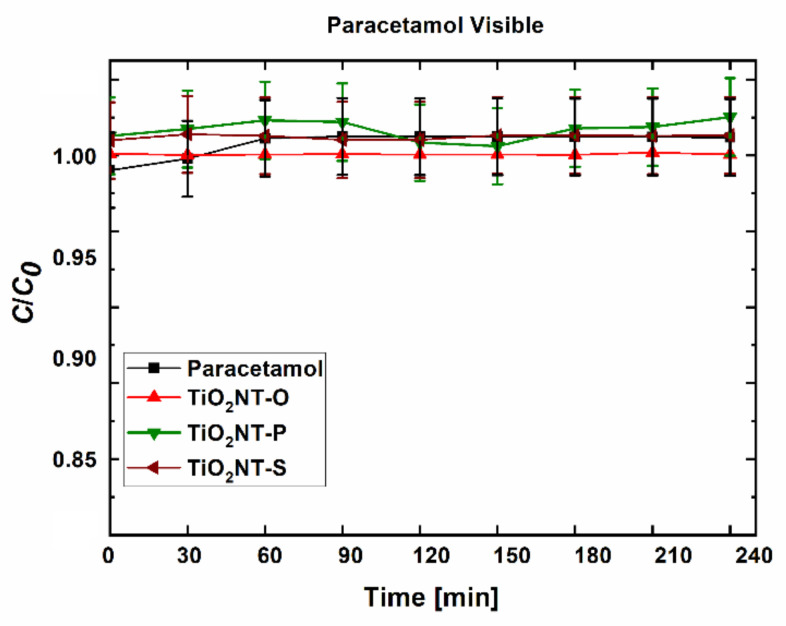
Paracetamol degradation under visible light.

The degradation of paracetamol under visible light was used to verify the dye sensitisation mechanism (Figure 11). Since paracetamol does not absorb in the visible region of the spectrum, any reduction in concentration can be attributed to photocatalysis [69]. No reduction in concentration was recorded for any of the materials when irradiated with visible light, thus, verifying the contribution of MB towards the apparent visible light activity. The MB molecule which has significant redox activity and obtains its colouration from its conjugated structure, can easily become decolourised when exposed to a photocatalyst. On the other hand, the paracetamol molecule either requires higher light intensities, or else it cannot be oxidised by visible light using the catalysts studied in this work.

**Figure 12:**
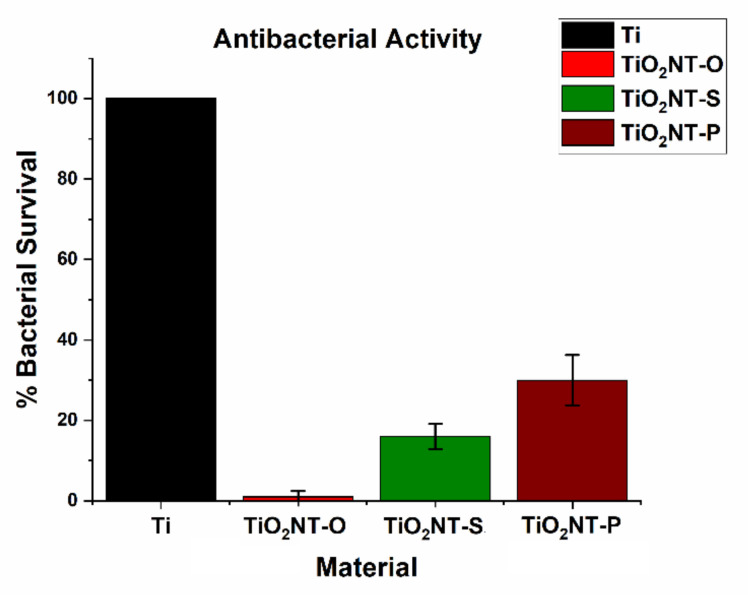
Effect of morphology on *E. coli* inactivation efficiency.

The photocatalytic disinfection mechanism differs from that of the degradation of organics since it is does not require the bacteria to be in contact with the photocatalyst surface. The reactive oxygen species (ROS) can migrate into the solution where they interact with the bacterial cell wall [70]. The ∙OH radicals, together with the other ROS produced, are thought to cause the peroxidation of the cell wall, inducing intracellular damage to the organelles and DNA. The electrons generated as part of the charge carrier pair can also enter the cell and cause reductive damage to the cell contents [71]. Despite the difference in mechanisms, Figure 12 shows that the bacterial inactivation mirrored the trend for the degradation of the organic molecules. Near complete inactivation was recorded for TiO_2_NT-O with a survival rate of only 1%. Survival rates of 16 and 30% were recorded for TiO_2_NT-S and TiO_2_NT-P, respectively. Although bacterial degradation could also occur by ROS migration, the efficiency of the arrays produced in the aqueous electrolyte was still quite low. This leads to the conclusion that the amount of charge carriers produced by TiO_2_NT-O is superior to that produced by the other materials. This is thought to result from better charge carrier separation. 

The mechanisms behind the photoinduced generation of charge carriers and the subsequent generation of ROS have been amply described in the literature [72,73,74]. In one of our previous works, the materials were ranked in terms of their ability to generate charge carrier pairs [75]. The radical ABTS and the cupric reducing antioxidant capacity (CUPRAC) assay were used to determine the number of electrons excited to the conduction band. The radical 2,2-diphenyl-1-picrylhydrazyl (DPPH) acts as a trap for other radicals, including ROS, such as the hydroxyl radical (∙OH). Thus, the ability of the surfaces to generate ROS can be compared. The TiO_2_NT-O surface was the most efficient in terms of both the electrons generated as well as the number of radical species produced. The TiO_2_NT-S and the TiO_2_NT-P surfaces placed second and third, respectively. The photocatalytic degradation tests carried out in this work, corroborate the findings of the earlier work, as the same order of reactivity was recorded. Thus, the TiO_2_NT-O surface was able to generate more charge carriers than the other two materials, and thus, the generation of a higher concentration of ROS. This resulted in the high activity of this material. 

## 4. Conclusions

The photocatalytic activity of three generations of different anodic nanotubes were compared based on their ability to degrade contaminants. All the synthesised materials were able to reduce the concentration of a variety of contaminants, both microbial and in the form of organic molecules. The effect of inorganic species on the photocatalytic activity was not evaluated in this study. These species might contribute to inhibition of the reactions involved in the photocatalytic process. Out of the materials investigated, the arrays belonging to the third generation, namely, TiO_2_NT-O, overall had a better photocatalytic performance. The combination of increased layer thickness, tube diameters and anatase fractions provided the basis for an efficient photocatalyst. The duration of the anodising process is also the shortest for TiO_2_NT-O, making its large-scale production more attractive.

## Figures and Tables

**Table 1:** Summary of the topological features of the nanotube arrays.

Material	Parameters	Layer Thickness (µm)	Tube Diameter (nm)	Wall Thickness (nm)	Aspect Ratio
TiO_2_NT-O	70 V, 1 h	9.99 ± 0.48	85–125	10.00 ± 2.00	79.92–117.52
TiO_2_NT-S	20 V, 6 h	1.45 ± 0.07	60–100	13.99 ± 2.00	14.5–24.17
TiO_2_NT-P	20 V, 3 h	0.68 ± 0.02	60–105	11.71 ± 2.00	6.48–11.33

**Table 2:** Spectral absorptance of the different nanotube arrays.

Material	Solar Absorptance (%)			
	UV (280–400 nm)	UV 365 nm	Visible (400–700 nm)	Total (280–700 nm)
TiO_2_NT-O	86.1 ± 1.3	86.7 ± 0.03	84.4 ± 1.2	84.6 ± 1.2
TiO_2_NT-S	84.9 ± 1.0	84.6 ± 0.08	79.9 ± 1.4	80.4 ± 1.3
TiO_2_NT-P	88.7 ± 1.0	88.6 ± 0.17	83.8 ± 1.1	84.3 ± 1.2
